# Evaluation of hydrophobic micro-zeolite-mixed matrix membrane and integrated with acetone–butanol–ethanol fermentation for enhanced butanol production

**DOI:** 10.1186/s13068-015-0288-x

**Published:** 2015-07-25

**Authors:** Chuang Xue, Decai Yang, Guangqing Du, Lijie Chen, Jiangang Ren, Fengwu Bai

**Affiliations:** School of Life Science and Biotechnology, Dalian University of Technology, Linggong Road 2, Dalian, 116024 China; School of Life Sciences and Biotechnology, Shanghai Jiao Tong University, Shanghai, 200240 China

**Keywords:** ABE fermentation, Pervaporation, Butanol recovery, Zeolite-mixed membrane

## Abstract

**Background:**

Butanol is regarded as an advanced biofuel that can be derived from renewable biomass. However, the main challenge for microbial butanol production is low butanol titer, yield and productivity, leading to intensive energy consumption in product recovery. Various alternative separation technologies such as extraction, adsorption and gas stripping, etc., could be integrated with acetone–butanol–ethanol (ABE) fermentation with improving butanol productivity, but their butanol selectivities are not satisfactory. The membrane-based pervaporation technology is recently attracting increasing attention since it has potentially desirable butanol selectivity.

**Results:**

The performance of the zeolite-mixed polydimethylsiloxane (PDMS) membranes were evaluated to recover butanol from butanol/water binary solution as well as fermentation broth in the integrated ABE fermentation system. The separation factor and butanol titer in permeate of the zeolite-mixed PDMS membrane were up to 33.0 and 334.6 g/L at 80°C, respectively, which increased with increasing zeolite loading weight in the membrane as well as feed temperature. The enhanced butanol separation factor was attributed to the hydrophobic zeolites with large pore size providing selective routes preferable for butanol permeation. In fed-batch fermentation incorporated with pervaporation, 54.9 g/L ABE (34.5 g/L butanol, 17.0 g/L acetone and 3.4 g/L ethanol) were produced from 172.3 g/L glucose. The overall butanol productivity and yield increased by 16.0 and 11.1%, respectively, which was attributed to the alleviated butanol inhibition by pervaporation and reassimilation of acids for ABE production. The zeolite-mixed membrane produced a highly concentrated condensate containing 169.6 g/L butanol or 253.3 g/L ABE, which after phase separation easily gave the final product containing >600 g/L butanol.

**Conclusions:**

Zeolite loading in the PDMS matrix was attributed to improving the pervaporative performance of the membrane, showing great potential to recover butanol with high purity. Therefore, this zeolite-mixed PDMS membrane had the potential to improve biobutanol production when integrating with ABE fermentation.

## Background

Butanol is considered as an advanced biofuel that can be derived from renewable biomass by ABE fermentation [1, 2]. However, the main challenge for butanol production by *Clostridium* spp. is low butanol titer, yield and productivity due to severe butanol toxicity to cells, leading to intensive energy consumption in product recovery [3, 4]. Therefore, various in situ butanol recovery technologies (such as adsorption, gas stripping and pervaporation) have been investigated with energy-efficient perspective since they could increase fermentation rate, mitigate butanol inhibition to cells by continuously recover butanol from fermentation broth [5, 6].

Pervaporation is recently attracting increasing attention, which allows selective removal of volatiles from model solution/fermentation broth through the membranes. In addition to the energy consumption from vacuum pump to create driving force for permeation, the phase change requires additional energy which should be at least equal to the heat of evaporation of the permeate. Therefore, it would be very energy-efficient if the membrane could permeate the target products with high selectivity. Compared to the conventional homogeneous polymeric membranes such as polysiloxane [7] and poly(1-trimethylsilyl-1-propene) (PTMSP) [8] etc., the PDMS membranes are more promising than others with excellent hydrophobicity as well as good chemical and mechanical stability [9, 10]. Therefore, the PDMS composite membranes have been evaluated its potential for butanol recovery by lots of scholars [11, 12]. Various hydrophobic zeolites had been used as filler in enhancing membrane selectivity for gaseous separation and organic solvent separation [13, 14]. It is known that ZSM-5 is a zeolite with medium pore size of <1 nm, high silicon-to-aluminum (Si/Al) ratio and super hydrophobicity. The zeolite-mixed PDMS membranes were once selected for product removal from ethanol/water, butanol/2,3-butanediol binary mixture due to their excellent hydrophobic nature and stability [15, 16]. Till now, there is little study about the zeolite-mixed PDMS membrane, especially ZSM-5 type of zeolite, for butanol or ABE recovery integrated with ABE fermentation.

In the present study, the homogeneous PDMS membrane and zeolite-mixed membranes were investigated to compare their performance on butanol recovery from butanol–water binary solution. The effect of zeolite loading weights on the membrane properties and pervaporation performance were investigated. Furthermore, the mixed membrane with the optimal loading weight of zeolite was directly integrated with ABE fermentation to remove ABE solvent from fermentation broth and to mitigate the inhibition of butanol to cells. The performance of this membrane was effective to enhance butanol production for integration of ABE fermentation to recover butanol. This integrated process with the pervaporative membrane also provides guidance for butanol or other bio-chemicals production by other bacteria.

## Results and discussion

### Characterization of zeolite-mixed PDMS membranes

**Figure 1:**
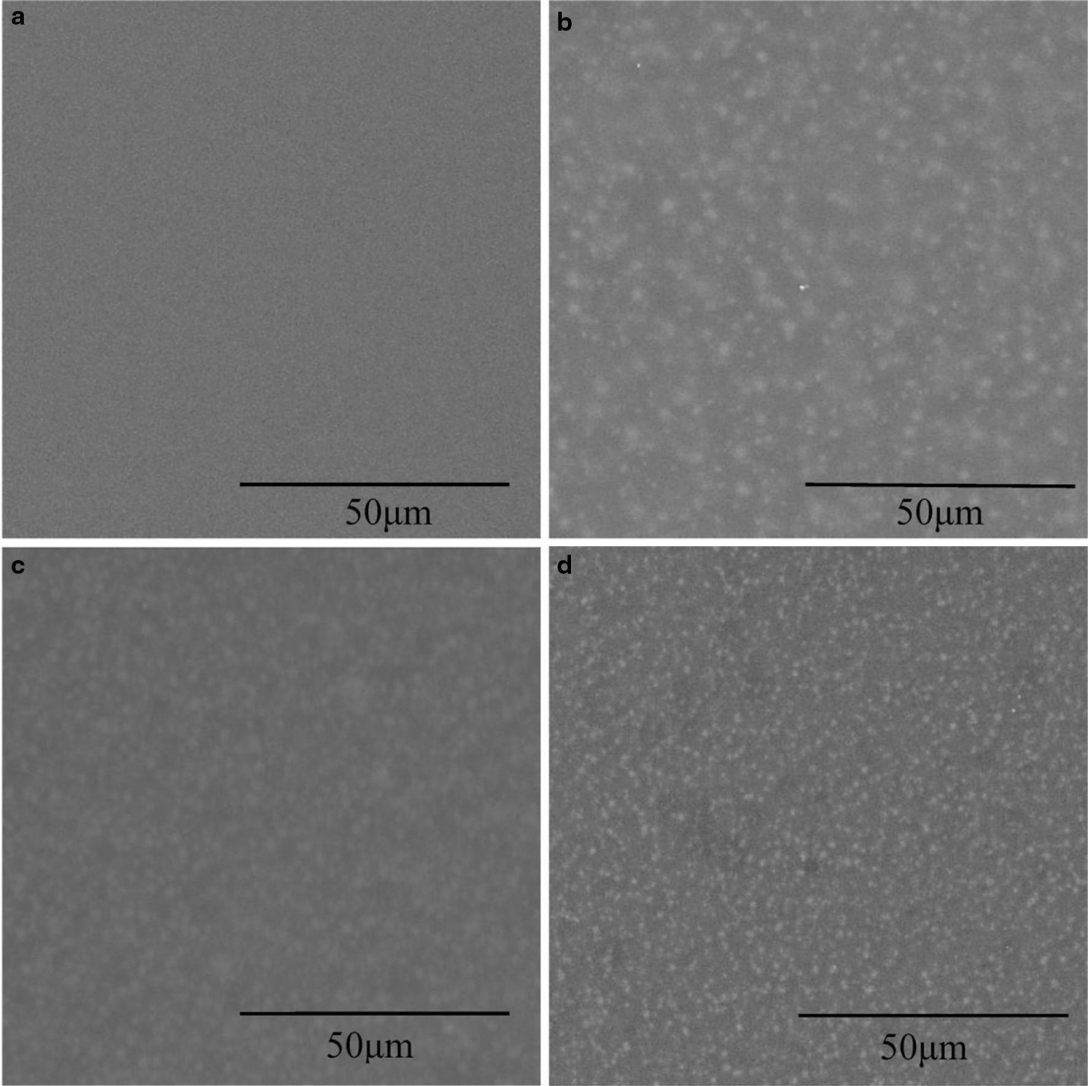
Scanning electron microscope (SEM) images of the PDMS and zeolite-mixed PDMS membrane. **a** No zeolite, **b** 20 wt%, **c** 50 wt%, **d** 80 wt%.

Scanning electron microscopy (SEM) images of the zeolite-mixed PDMS membranes with different zeolite loading weights were illustrated in Figure 1. As seen in Figure 1, it appeared that the zeolite particles had a good interface compatibility with the hydrophobic PDMS. With sonication treatment, the dispersion of zeolite particles could be uniformly in the PDMS membrane matrix. This can be attributed to the hydrophobic nature of the zeolite particles, their favorable association with the prior dispersed silicone elastomeric base. Due to this structural integrity, the mixed matrix membrane can be observed as single composite matrix, and the performance of the membrane can be thus evaluated by changing loading weights of the zeolites in the membrane matrix.

In addition, it is very critical to get the particles well distributed in the PDMS matrix. In case particles agglomerate, the leaky flow would dominate the mass transport across the agglomerate regions, and the particles would lost their abilities in improving the membrane selectivity. Fortunately, the dispersed zeolite particles in the membranes are intimately enclosed by the surrounding PDMS matrix, guaranteeing the permeate flow to be molecularly selective.

### The effect of zeolite loading on the membrane performance

The performance of PDMS membranes with different loading weights of zeolites were compared in Table 1. In general, butanol titer in permeate and separation factor increased with increasing zeolite loading weight in the PDMS polymer, which was attributed to improved hydrophobicity of the membrane that limited water transport. When zeolite loading weight increased from 0 to 80 wt% at 37°C, butanol titer in permeate and separation factor increased from 101.6 g/L and 7.4 to 203.1 g/L and 16.7, respectively. However, total flux through the membrane decreased by ~40% with increasing the zeolite loading to 80 wt%, indicating the membrane swelling was suppressed with more fillers incorporated into the PDMS matrix. As known previously, zeolite-filled membranes showed the reduced permeability for all the permeating components, accordingly, the decreased membrane fluxes were observed for butanol/2,3-butanediol with the increased zeolite loading weight in the membrane [15]. The stable structure of zeolite is usually not susceptible to membrane swelling which occurs in the zeolite regions of the filled membranes. The PDMS polymer chains near the zeolite regions may be restrained by the interfacial interactions. Therefore, the zeolite particles act as the physical cross-linker stations for the surrounding polymer chains.

**Table 1:** Comparison of pervaporation performance of the homogeneous PDMS membrane and zeolite-mixed membranes

	No zeolite		20 wt% zeolite		50 wt% zeolite		80 wt% zeolite	
	37°C	80°C	37°C	80°C	37°C	80°C	37°C	80°C
Total flux, g/m^2^ h	160.3	435.4	152.4	413.6	133.6	386.2	99.8	377.2
Butanol flux, g/m^2^ h	16.3	96.4	18.5	96.0	22.1	106.3	20.3	126.2
Butanol titer in permeate, g/L	101.6	221.4	121.5	232.1	165.2	275.3	203.1	334.6
Separation factor	7.4	18.7	9.1	19.8	13.0	24.9	16.7	33.0

Interestingly, the zeolite-mixed membranes displayed higher butanol separation factor for permeable butanol, and 80 wt% zeolite loading gave the highest butanol selectivity over water. It should be noted that the hydrophobic nature of zeolite is crucial for the improved butanol selectivity. However, it was difficult to obtain defect-free PDMS membrane when higher weights of zeolite (e.g., 100 wt%) were loaded in the membrane. From butanol purity point of view, it was thus suggested that optimal zeolite loading in the PDMS membrane was around 80 wt% in the mixed PDMS membrane.

### Effects of operating temperatures in feed

Feed temperature is one of key parameters to influence the pervaporation performance. Table 1 shows the effect of feed temperature (37 and 80°C) on pervaporation performance in a feed solution containing 15.0 g/L butanol. When the feed temperature increased from 30 to 80°C, both total and butanol flux sharply increased, due to the increased diffusion of the permeating molecules, as well as the increase of desorption rate of butanol in zeolite/PDMS matrix. Butanol titer in permeate and separation factor also increased with increasing the temperature and achieved the maximum of 334.6 g/L and 33.0 at 80 wt% loading of zeolite, respectively. The increased temperature would produce more free volumes in polymer chains to facilitate the permeation of the compounds. The demonstrating data suggested that the hydrophobic channels of zeolite made a considerable contribution to the selective permeation of butanol molecules and the diffusion of the solution.

Based on the demonstrating data above, it is generally impossible for this type of zeolite-mixed membranes to obtain simultaneously both the enhanced mass flux and selectivity. The tradeoff between permeability and selectivity is valuable for membrane separation. In consideration of butanol purity, the selectivity of the membrane is of foremost importance, and the permeability or flux could be made satisfactory by fabricating the thin or ultrathin membrane. Since the 80 wt% zeolite-mixed membrane has the highest selectivity for butanol, the fed-batch ABE fermentation integrated with this membrane was used to further investigate its performance for butanol recovery.

### Enhanced ABE fermentation with the zeolite-mixed membrane

The ABE fermentation without/with pervaporation was carried out to investigate the performance of product recovery from active fermentation broth. In typical batch fermentation without pervaporation, with the medium initially containing 80.0 g/L glucose, about 12.8 g/L butanol, 6.0 g/L acetone, and 1.9 g/L ethanol were produced when the fermentation ended at ~52 h with about 9.5 g/L glucose remaining in the medium (Table 2). The declined cell density was observed at the end of fermentation, indicating that butanol led to cell autolysis due to serious toxicity to cell. Acetic and butyric acids were produced during the first 24 h, and then these acids were assimilated by cells for ABE production in solventogenesis. The final acetic and butyric acids were 3.6 and 3.1 g/L, respectively. The butanol and ABE yields from glucose were 0.18 and 0.29 g/g, respectively. The butanol and ABE productivity was 0.25 and 0.40 g/L h, respectively.

**Table 2:** Comparison of ABE fermentations by *C. acetobutylicum* ATCC55025 without/with pervaporation

Fermentation parameters	Batch fermentation	Fed-batch fermentation with pervaporation
Initial glucose, g/L	80.0 ± 1.5	80.0 ± 1.1
Consumed glucose, g/L	70.5 ± 2.0	172.3 ± 1.6
Residual glucose, g/L	9.5 ± 0.5	10.0 ± 0.5
Maximum OD	3.90	5.50
Fermentation time, h	52	120
Acetone, g/L	6.0 ± 0.1	17.0 ± 0.6
Butanol, g/L	12.8 ± 0.9	34.5 ± 1.5
Ethanol, g/L	1.9 ± 0.2	3.4 ± 0.2
Total ABE, g/L	20.7 ± 1.2	54.9 ± 1.5
Butanol yield, g/g	0.18 ± 0.02	0.20 ± 0.01
ABE yield, g/g	0.29 ± 0.03	0.32 ± 0.01
Butanol productivity, g/L h	0.25 ± 0.02	0.29 ± 0.02
ABE productivity, g/L h	0.40 ± 0.02	0.46 ± 0.02
Acetic acid, g/L	3.6 ± 0.2	2.7 ± 0.1
Butyric acid, g/L	3.1 ± 0.2	3.2 ± 0.1
Total acids, g/L	6.7 ± 0.4	5.9 ± 0.2

**Figure 2:**
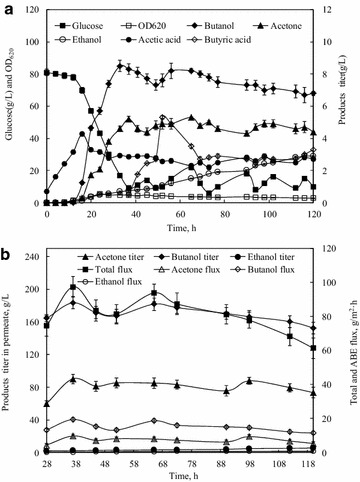
ABE fermentation by pervaporation with the zeolite-mixed PDMS membrane. **a** Kinetics of products and glucose concentrations in the fermentation broth, **b** pervaporation performance of the membrane during ABE fermentation.

The time course of fed-batch ABE fermentation incorporating the zeolite-mixed membrane is shown in Figure 2a. As shown in Figure 2a, the ABE fermentation was initiated with P2 medium containing ~80.0 g/L glucose, and then ABE and acids were produced gradually with time. When glucose was reduced to 9.0 g/L at 37 h, the fed-batch medium containing ~400 g/L glucose was fed into the bioreactor intermittently until the end of fermentation. When the butanol titer increased to 5.7 g/L, the pervaporation was started at 24 h. As seen in Figure 2a, from 28 h to the end of the fermentation, due to the removal of products from the fermentation broth by pervaporation, butanol, acetone and ethanol titer in the fermentation broth were relatively stable in the range of 6.7–8.5, 4.4–5.3 and 0.6–2.9 g/L. The demonstrating results above indicated that the in situ product recovery with the zeolite-mixed membrane could make the products titer in fermentation broth within the stable ranges by the continuous removal of ABE solvents.

As shown in Table 2, the fed-batch fermentation with pervaporation could produce 34.5 g/L butanol, 17.0 g/L acetone and 3.4 g/L ethanol, with 172.3 g/L glucose consumed in 120 h. The butanol and ABE productivities were 0.29 and 0.46 g/L h, respectively, increasing by 16.0 and 15.0% as compared to batch fermentation without pervaporation. The overall butanol and ABE yields from glucose were 0.20 and 0.32 g/g, and both were ~10% higher than those in batch fermentation, which could be attributed to the enhanced reassimilation of acetic and butyric acids by the cells in solventogenesis, instead of being accumulated. Clearly, the removal of butanol by pervaporation not only alleviated butanol toxicity, but also increased fermentation rate and butanol yield.

### In situ product recovery with the zeolite-mixed membrane

The pervaporation with the 80 wt% zeolite-mixed membrane in fed-batch ABE fermentation was carried out to investigate the performance of product recovery from active fermentation broth. The pervaporation performance of the membrane during ABE fermentation is shown in Figure 2b. After pervaporation started, butanol titer, acetone titer and ethanol titer in permeate maintained at steady levels with the range of 152.8–183.5, 60.2–90.3 and 0.1–0.8 g/L, respectively. The total flux and butanol flux were also stable in the limited range of 61.4–97.5 and 11.4–19.4 g/m^2^ h, respectively. The steady pervaporation performance of the membrane was attributed to the stable ABE titer in fermentation broth as well as the excellent hydrophobic nature of the zeolite/PDMS matrix.

The solubility of butanol in water at 20°C is 7.7% (w/w) and thus phase separation occurs spontaneously when butanol concentration is higher than 8%. During the overall pervaporation process, the zeolite-mixed membrane produced a highly concentrated condensate containing 169.6 g/L butanol or 253.3 g/L ABE. The butanol concentration in the condensate was high enough for effective phase separation, generating an organic phase containing >600 g/L butanol. When butanol is concentrated to such a high titer, energy consumption in final product purification as fuel or solvent by existing dewatering technologies can be reduced dramatically.

The stability of 80 wt% zeolite-mixed PDMS membrane for ABE recovery from fermentation broth was investigated for >200 h, and the results showed stable performance with a little fluctuation in butanol separation factor and mass flux for more than 20 samples during the tested period. There was no obvious decrease in mass fluxes, indicating no fouling or clogging of the membrane. Since the membrane surface was smooth and nonporous (Figure 1), the cells that tended to induce membrane fouling were difficult to stick to or block the membrane under the cross-flow conditions. Therefore, the zeolite loading PDMS membrane was stable and could keep a long lifetime for butanol recovery from ABE fermentation broth.

### Comparison to other membranes with pervaporation

Pervaporation with various kinds of membranes has been widely studied by lots of scholars. In general, the pervaporation performance for butanol recovery was subjected to operation conditions such as feed temperature, feed butanol titer, vacuum pressure, membrane thickness and materials, etc. The porous membranes such as polypropylene (PP) and polyvinylidene fluoride (PVDF) materials had high mass flux, easily allowing water permeation through the membranes, which was not desirable to recover the product with high butanol titer [17, 18].

The PDMS membranes have been considered as the promising membranes for butanol recovery with excellent mass permeation. The silicalite-1/PDMS membrane had high total flux of 600–700 g/m^2^ h and butanol separation factor of 90–100, respectively, possessing both the advantages of inorganic and organic membranes [19, 20]. The tri-layer PDMS/PE/Brass membrane could recover butanol from butanol–water solution with the total flux of 132 g/m^2^ h and separation factor of 32 [10]. In addition, it was reported that the ultra-thin MFI zeolite membrane had super high total flux of about 4,000 g/m^2^ h and separation factor of 10 [21]. Furthermore, the hydrophobic property of this zeolite membrane could be controlled by altering the silicon-to-aluminum (Si/Al) ratio of the zeolite. However, since it is generally impossible to obtain both the enhanced permeability and selectivity, the choice between permeability and selectivity should be made for effective membrane separation. More importantly, none of the above-mentioned membranes were tested for butanol recovery in the integrated ABE fermentation system.

The pervaporations integrated with ABE fermentation have been recently investigated due to the increasing attention to butanol dehydration for biobutanol production. The butanol titers in permeate and separation factor were only 46.5 g/L and 7.0, respectively, using the homogeneous PDMS membrane for pervaporation during ABE fermentation, which were not satisfactory due to the limited hydrophobicity of the PDMS polymer [12]. The PDMS/ceramic composite membrane could produce the condensate containing butanol titer in permeate of 81.2–118.0 g/L and butanol separation factor of 5.1–27.1 when in situ removing ABE solvent from fermentation broth [22]. In our recent study, the asymmetric PDMS/PVDF composite membrane was fabricated to recover ABE solvent from fermentation broth in the integrated fermentation system [23]. This membrane produced the condensate containing butanol titer of 139.9–154.0 g/L and ABE titer of 252.2–266.9 g/L. The present study indicated that the zeolite-mixed membrane incorporated with fed-batch ABE fermentation could stably recover the product containing butanol titer of 152.8–181.9 g/L and ABE titer of 226.7–276.1 g/L, with the increased overall butanol productivity and yield by 16.0 and 11.1%, respectively. The continuous removal of butanol from fermentation broth by pervaporation could alleviate butanol inhibition to cells and contribute to the assimilation of organic acids for ABE production by active cells. Zeolite loading in the PDMS matrix was attributed to improving the pervaporative performance of the membrane, showing great potential to recover butanol with high purity. Moreover, pervaporation was considered as an energy-efficient process with energy consumption of <10 MJ/kg butanol, which had been discussed by our recent publication [6]. For scaling up this process, the membrane fouling by the adsorption and infiltration of cells and macromolecules is the main concern, which may lead to the decreased membrane flux and selectivity. The membrane could be restored by water cleaning, but the downtime would be increased significantly. In addition, increasing the feed flow rate or the smoothness of the membrane surface would be effective to avoid the membrane contamination by decreasing the risk of cells or macromolecules adsorbed on the surface of the membrane. Therefore, the pervaporation of the zeolite-mixed PDMS membrane integrated with ABE fermentation had a great potential applied in commercialized butanol production.

## Conclusions

The zeolite-mixed PDMS membrane was so effective that the butanol titer in permeate and separation factor could be achieved to 334.6 g/L and 33.0 at 80°C, respectively, which was much better than those of the PDMS membrane without zeolite loading. In fed-batch fermentation incorporated with the 80 wt% zeolite-mixed membrane, it could improve the butanol (ABE) productivity and yield, recovering a highly concentrated condensate containing 169.6 g/L butanol (253.3 g/L ABE) from active fermentation broth with stable mass flux. It is thus desirable to use the zeolite-mixed PDMS membrane for efficient butanol recovery in biobutanol production.

## Methods

### Culture and media

*Clostridium acetobutylicum* ATCC 55025 was used in this study. The seed culture was prepared in the Clostridial growth medium (CGM) containing 30 g/L glucose, 2 g/L yeast extract, 1 g/L tryptone, minerals, and vitamins in a phosphate buffer as described in Xue et al. [2], and incubated at 37°C for ~16 h until active growth was observed. ABE fermentation was studied using the P2 medium containing: glucose (80 g/L), yeast extract (1 g/L), KH_2_PO_4_ (0.5 g/L), K_2_HPO_4_ (0.5 g/L), ammonium acetate (2.2 g/L), vitamins (1 mg/L para-amino-benzoic acid, 1 mg/L thiamin and 0.01 mg/L biotin), and mineral salts (0.2 g/L MgSO_4_∙7H_2_O, 0.01 g/L MnSO_4_∙H_2_O, 0.01 g/L FeSO_4_∙7H_2_O, 0.01 g/L NaCl), prepared according to the procedures described previously [24]. The media were sterilized by autoclaving at 121°C and 15 psig for 30 min. All solutions were purged with nitrogen for 1 h through a sterile 0.2 μm filter, either before or after autoclaving.

### Fed-batch fermentation and pervaporation start-up

**Figure 3:**
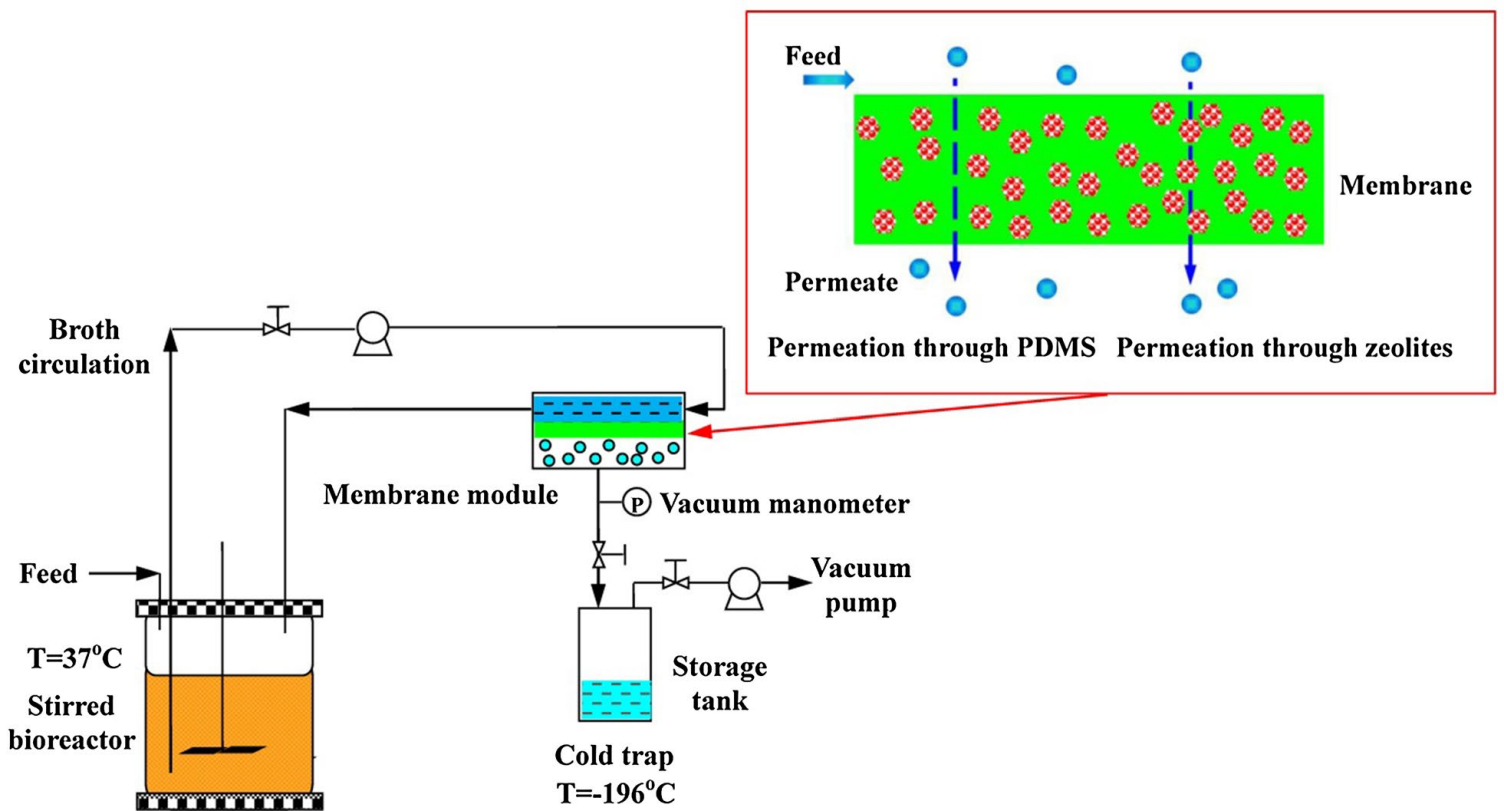
Experimental setup for ABE fermentation with pervaporation and butanol permeation assisted by zeolites through the membrane.

The mini-type bioreactor containing 0.2 L of the P2 medium was inoculated with 20 mL of growing cells (~16 h) and then maintained at 37°C and pH 5.0, by the addition of 2 N NH_4_OH, and agitated at 150 rpm for 24 h until butanol titer in the fermentation broth was over 5.0 g/L. Then, the pervaporation with the micro-zeolite-mixed PDMS membrane was initiated to continuously recover ABE solvent from active fermentation broth until the end of the fermentation. When glucose was decreased to <10.0 g/L, the concentrated medium containing ~400.0 g/L glucose was fed into the bioreactor till the fermentation ended. The integrated fermentation system with pervaporation for in situ product recovery was illustrated in Figure 3. Liquid samples were withdrawn from the bioreactor and storage tank periodically for the analysis of glucose, fermentation products and recovered products.

### Preparation of the homogeneous PDMS and micro-zeolite-mixed PDMS membrane

For the PDMS membrane fabrication, the base solution from the Sylgard^®^184 silicone elastomer kit (Dow Corning, USA) was mixed with the curing agent in the ratio of 10:1 using pentane as the solvent to dilute the mixture. The mixture was stirred completely for 5 min and then 8,000×*g* centrifuged for 5 min to wipe off air bubble. The mixture was placed on a cleaning glass plate and cast evenly using a micron film applicator (Paul N. Gardner Company, USA). The mixture on the glass plate was then heated in oven for 3 h at 100°C. After the membrane cure, the membrane was carefully peeled off for pervaporation.

The zeolite particles named CBV28014 (ZSM-5 type of the zeolite) were purchased from Zeolyst International (USA), with surface area of 400 m^2^/g. The micro-zeolite-mixed PDMS membrane was fabricated with the zeolite particles incorporating into the PDMS polymer. The base solution and curing agent in the ratio of 10:1 was mixed using pentane as the solvent, and zeolite with a mass ratio of 20, 50 and 80 wt% were dispersed into the solution, respectively. Firstly, the pre-dried zeolite particles were added to the prepared 10 wt% silicone elastomeric base solution in pentane followed by vigorous manual mixing. The mixture was then added to the curing agent and ultrasonic processed in a sonication bath for 30 min to disperse the particles in the PDMS polymer. The mixture was uniformly coated on a clean glass plate with a micron film applicator and then placed in vacuum to degas. After heating at 100°C for 3 h to cure the membrane, the membrane was peeled off the glass plate. The thickness of the PDMS and zeolite-mixed PDMS membranes was 100 μm. The effective area of the PDMS and zeolite-mixed PDMS membranes were 58 cm^2^. The scanning electron microscopy (SEM) (Quanta450, FEI, USA) was used to analyze the PDMS and zeolite-mixed PDMS membranes morphologies and the images were shown in Figure 1.

### Pervaporation with the PDMS membrane and zeolite-mixed PDMS membrane

The butanol–water solution containing ~15.0 g/L butanol was used to investigate the pervaporation performance of the membranes at the designated temperature. The feed solution was circulated at a flow rate of 1.2 L/min to minimize the boundary layer thickness and maximize mass transfer. Vacuum was provided on the downstream side of the membrane using a vacuum pump with <1 kPa as the driving force. The recovered permeate was collected in the storage tank immersed in liquid nitrogen.

The flux (ABE and total) and separation factor (SF) were calculated as follows:

$$ \text{Flux} = \frac{W}{At} $$

$$ \text{SF} = \tfrac{y/(1 - y)}{x/(1 - x)} $$

where *W* is the weight of the recovered permeate in g, *A* is the membrane area in m^2^ and *t* is the time (h) for the sample collection. *x* and *y* is the weight fractions of components in the feed and permeate samples in the pervaporation, respectively.

### Analytical methods

Glucose and products in the fermentation broth were assayed after removing cells by centrifugation at 10,000×*g* for 5 min. The glucose concentration was determined with a glucose analyzer (Biosensor SBA-50, Institute of Biology, Shandong Academy of Sciences, Shandong, China). Butanol, acetone and ethanol were determined using a gas chromatograph (Agilent 6890A GC) equipped with a hydrogen flame ionization detector (FID), following the method previously described [23]. Acetic acid and butyric acid were analyzed using the HPLC system (Waters 1525) equipped with an organic acid analysis column (Aminex HPX-87H, 300 mm × 7.8 mm) and also described in our previous study [23].

## Abbreviations

ABE: acetone–butanol–ethanol
PDMS: polydimethylsiloxane
PTMSP: 1-trimethylsilyl-1-propene
SEM: scanning electron microscopy
PP: polypropylene
PVDF: polyvinylidene fluoride
CGM: Clostridial growth medium
